# Assessing Mental Health and Emotional Well-Being in Tunisian Musicians

**DOI:** 10.1192/j.eurpsy.2026.11243

**Published:** 2026-07-31

**Authors:** A. Aloui, I. Kacem, N. Ganoun, A. chouchane, E. Toulgui, M. Bouhoula, M. Makhloufi, H. Kalboussi, O. El Maalel, S. Chatti

**Affiliations:** 1 Department of Occupational Medicine, Farhat Hached University Hospital; 2 Physical Medecine and Rehabilitation Department, Saloul University Hospital, Sousse, Tunisia

## Abstract

**Introduction:**

Musicians are exposed to unique stressors related to artistic practice, performances, and professional demands. These pressures can impact their mental health and emotional well-being. Understanding musicians’ psychological status is essential for developing appropriate support strategies.

**Objectives:**

To describe the mental health status of the musicians and its characteristics

**Methods:**

This is a cross-sectional study among music academy students from the Music Institute of Sousse and Orchestra Musicians. A self-administrated questionnaire was distributed either manually or via e-mail to collect information about the personal and occupational history of participants. The mentel health was assesed by the the mental component score (MCS-12) of the the Short Form Survey SF-12.

**Results:**

Eighty-one questionnaires were included. The age of musicians ranged from 18 to 62 years (mean 25.19,±8.534).The sample consists of 45 men (56.2%) and 35 women (43.8%) with a sex ratio of 1.28. Among 81 musicians, 35(44%) were orchestra musicians, 21(26%) were academy students from different classes starting 1st year to 2nd master degree class and 25(31%) had both statuses. Most of the participants were string musicians 62 (76.6%) followed by 7wind/brass musicians (8.6%) 7 percussion musicians (8.6%) and 5 keyboard musicians (6.2%).The mean MCS12 in our study population was 45.22 (10.23SD) with scores ranging from14.59 to 58.74. Results have revealed that 23 musicians (28.39%) had an MCS-12 lower than the reference value. Male musicians had a better MCS-12 mean compared to female musicians with a slight difference between academy students and orchestra musicians . Keyboard musicians had the lowest MCS-12 mean (39.97, 14.20 SD) which is a score underneath the reference value indicating in this case a low mental health functioning. Percussionists had the highest MCS-12 mean indicating better health functioning in comparison with other instruments.

**Conclusions:**

Mental health issues among musicians represent a significant concern, reflecting both occupational stressors and creative demands. Targeted preventive strategies and supportive interventions are essential to promote psychological well-being in this population.

**Disclosure of Interest:**

None Declared

